# A Functional Interplay between IGF-1 and Adiponectin

**DOI:** 10.3390/ijms18102145

**Published:** 2017-10-14

**Authors:** Stefania Orrù, Ersilia Nigro, Annalisa Mandola, Andreina Alfieri, Pasqualina Buono, Aurora Daniele, Annamaria Mancini, Esther Imperlini

**Affiliations:** 1 Dipartimento di Scienze Motorie e del Benessere, Università degli Studi di Napoli “Parthenope”, via Medina 40, 80133 Napoli, Italy; orru@uniparthenope.it (S.O.); annalisa.mandola@uniparthenope.it (A.Md.); andreina.alfieri@uniparthenope.it (A.A.); pasqualina.buono@uniparthenope.it (P.B.); 2 IRCCS SDN, via E. Gianturco 113, 80142 Napoli, Italy; 3 CEINGE-Biotecnologie Avanzate S.c.a r.l., Via G. Salvatore 486, 80145 Napoli, Italy; nigro@ceinge.unina.it (E.N.); aurora.daniele@unicampania.it (A.D.); 4 Dipartimento di Medicina e di Scienze della Salute “Vincenzo Tiberio” Università degli Studi del Molise, Campobasso, Italy; 5 Dipartimento di Scienze e Tecnologie Ambientali Biologiche Farmaceutiche, Università della Campania “Luigi Vanvitelli”, Via G. Vivaldi 42, 81100 Caserta, Italy

**Keywords:** insulin-like growth factor-1 (IGF-1), growth hormone (GH), adiponectin, obesity, diabetes, insulin resistance, heart failure, cardiovascular disease, cancer

## Abstract

A functional relationship is suggested between two well-known protein hormones, insulin-like growth factor 1 (IGF-1) and adiponectin. In the last two decades in fact, different experimental evidence has indicated a non-random link between them. Here, we describe briefly the IGF-1 and adiponectin systems, and we then focus on their putative interplay in relation to several pathological conditions, including obesity, diabetes, insulin resistance, cardiovascular disease, and cancer. Although the existing studies are hardly comparable, they definitely indicate a functional connection between these two protein hormones. In conclusion, the current knowledge strongly encourages further research into the common, as well as novel, mechanisms through which IGF-1 and adiponectin exert their concerted action.

## 1. Introduction

Insulin-like growth factor-1 (IGF-1) and adiponectin belong to a class of protein hormones, and their specific mechanisms of action, target tissues/organs, and signaling have been investigated for a long time [1,2]. In the last two decades, experimental evidence has suggested a link between IGF-1 and adiponectin, specifically in tissues carrying both IGF-1 and adiponectin receptors. Here, we review the current understanding of the presumptive concerted action between these two protein hormones. To reach this goal, we provide a concise description of both IGF-1 and adiponectin systems, and we focus on the putative relationship of the two hormones in obesity, diabetes, insulin resistance, heart failure, and cancer.

## 2. Insulin-like growth factor-1 (IGF-1)

The protein hormone IGF-1 (originally named somatomedin C) is a 70-amino acid tissue growth factor mainly produced by the liver following growth hormone (GH) stimulus [3]. In turn, IGF-1 mediates many actions of GH, such as anabolic functions and growth-promoting effects, exhibits mitogenic and insulin-like metabolic activities [4], and negatively modulates GH [5]. In healthy subjects, other tissues secrete IGF-1 in response to the paracrine/autocrine action of GH [3,6]; moreover, its expression is also influenced by estrogens, adrenocorticotropic hormone (ACTH), thyrotropin releasing hormone (TRH), luteinizing hormone (LH), follicle-stimulating hormone (FSH), human chorionic gonadotropin (HCG), insulin, and other GFs, e.g., platelet-derived growth factor (PDGF), epidermal growth factor (EGF), and fibroblast growth factors (FGFs) as well as age, sex, diet, and nutrition [5].

**Figure 1:**
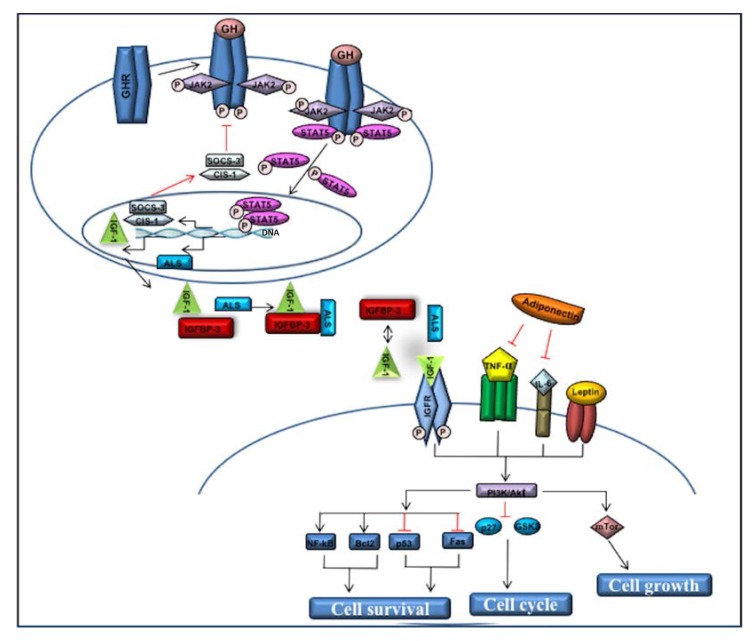
Growth hormone (GH)/insulin-like growth factor 1 (IGF-1) axis via JAK2-STAT5 signaling pathway leads to IGF-1 transcription/translation and modulates cell survival, cell cycle, and cell growth, also through the adiponectin action. Fas: cell surface death receptor; GSK3: glycogen synthase kinase 3.

The complex mechanism through which GH stimulates IGF-1 release is now well known (Figure 1). GH hormone binds to the extracellular domain of its receptor (GHR), present on the cell surface of different target tissues; this binding induces the autophosphorylation of the intracellular protein JAK2, which, in turn, together with the activated GHR, induces STAT5 phosphorylation. Successively, p-STAT5 dimers and translocates into the nucleus, where it determines the upregulation of several gene targets, including IGF-1 and the acid-labile subunit (ALS), a leucine-rich glycoprotein [7]. This pathway can be inhibited by postreceptor signaling inhibitors, such as members of the suppressor of cytokine signaling family (CIS/SOCS): the GH itself rapidly triggers the expression of SOCS-3 and Cis-1 in the liver and these proteins, acting in a negative feedback mechanism, are able to block STAT5 phosphorylation. Moreover, SOCS-3 inhibits JAK2 through a different mechanism that requires the presence of GHR [8].

IGF-1 shows sequence homology to insulin and IGF-2, another growth factor secreted by hepatocytes predominantly in the fetal age [9]; furthermore, IGF-1 concentrations gradually increase in childhood and puberty, while they decrease during aging [10].

The human *igf-1* gene is located on chromosome 12 and can be alternatively spliced into multiple transcripts, encoding both circulating IGF-1 and tissue-specific isoforms (IGF1Ea–c); the IGF1Ec isoform, known as Mechano Growth Factor, is strongly upregulated in the skeletal muscle following mechanical loading [11,12,13].

Circulating IGF-1 is associated with IGF-binding proteins (IGFBPs), which regulate hormone activity, by increasing its half-life and modulating the binding to the receptor [3,7,14,15]. The IGFBP family consists of six members (IGFBP-1–6), with IGFBP-3 carrying 75% or more of serum IGF-1 and IGF-2 in hetero-trimeric complexes that also contain ALS [7]. In order to interact with its receptor, IGF-1 dissociates from IGFBPs via an enzymatic process [7].

The endocrine, autocrine, and paracrine functions of IGF-1 are mediated through the binding to the type 1 IGF receptor (IGF-1R), a tyrosine kinase membrane receptor, which is ubiquitously expressed and exists as a homodimer and as a heterodimer in association with insulin receptor isoforms A or B [4,9]. The IGF-1-mediated signal transduction leads to the phosphorylation of insulin receptor substrates (IRS) and Shc proteins and the activation of PI-3K/Akt and Ras-MAPK signaling pathways, thus enhancing cellular proliferation, survival, angiogenesis, and invasion [5].

The GH/IGF-1 axis acts on growth, development, and differentiation in several tissues [16]; it also affects glucose and lipid metabolism, by switching fuel utilization toward fat oxidation [17,18,19,20]. Due to its anabolic properties, IGF-1, along with GH, is one of the most abused doping agents in sport competitions and, hence, they are both annually included in the World Anti-Doping Agency (WADA) list of banned drugs [3,21]. Controversial results describe the role in GH/IGF-1 in gaining muscle strength, in promoting muscle protein synthesis, in increasing fatty acid availability, and in sparing of glycogen stores [22,23,24], whereas the molecular effects of supraphysiological doses of IGF-1 on primary cultures of human lymphocytes clearly show their negative impact on health [25,26,27]. Nevertheless, GH and/or IGF-1 are assumed, at supraphysiological concentrations, alone or in combination with other doping agents [21].

There are, of course, pathological conditions that affect GH secretion and, hence, circulating levels of IGF-1. GH deficiency (GHD) is an endocrine disease, characterized by a reduced production of GH, and showing different clinical features, according to the age of onset, the major being the growth failure [28,29]. GHD determines several adverse effects, which include abnormalities in cardiac size and function as well as changes in body fat mass and distribution [28,30], thus leading to a phenotype linked to insulin resistance and glucose intolerance [31]. Acromegaly is also a rare endocrine syndrome, characterized by an excessive secretion of GH, commonly caused by pituitary adenoma [32]. The long-term exposition to high levels of GH/IGF-1 determines a wide range of dysfunctions, including cardiovascular and metabolic impairments [32,33,34].

## 3. Adiponectin

Among cytokines produced and secreted by adipose tissue, adiponectin is the most abundant adipokine [35]. Structurally, adiponectin is a 28 kDa protein constituted by 244 amino acids; three domains can be distinguished: (1) the N-terminal variable region with a great divergence between species; (2) the collagenous domain, named so because of its homology with collagen; and (3) the C-terminal globular domain, which represents the receptor-binding region. Adiponectin monomer assembles in complexes of different molecular weights: low-molecular-weight (LMW), medium-molecular-weight (MMW), and high-molecular-weight oligomers (HMW) [35].

Physiologically, adiponectin circulates in concentrations between 5 and 30 μg/mL, representing approximately 0.01% of all plasma proteins. Although specifically produced only by adipose tissue, adiponectin acts through the binding of widely expressed receptors, namely AdipoR1 and AdipoR2. AdipoRs are 7TM receptors with the C-terminus inside the cells and the N-terminus outside [36]. T-cadherin has been successively discovered as a third receptor for adiponectin with a higher affinity for the hexameric and HMW oligomers [37,38]. After the recruitment of several adaptor proteins, adiponectin receptors may activate many different molecular pathways. Several factors may be taken into account, namely: (i) metabolic conditions (adiponectin is considered a starvation hormone under fasting conditions, stimulating food intake decreasing energy expenditure, and promoting fat storage); (ii) environment, in terms of inflammatory and stress oxidative molecules; (iii) tissues or organs considered; and (iv) immune system activation or suppression. T-cadherin lacks an intracellular domain, so the specific biologic function due to the adiponectin–T-cadherin interaction remains unknown. Adiponectin may exert multiple effects on various tissues and organs, based on its oligomeric isoforms, pathophysiological status, and targeted cells/tissues [35,39,40]. One of the crucial effects of adiponectin is its regulation of glucose and lipid metabolism [41]. Overall, the protective effects of adiponectin against inflammation and insulin resistance are due to its capacity to ameliorate lipid and simple carbohydrate profiles [42]. Adiponectin is known to modulate vascular remodelling and suppress endothelial cell migration and adhesion [43]. Additionally, adiponectin plays a key role in bone homeostasis [44].

Reduced adiponectin levels [45,46,47,48,49] and/or reduced HMW/LMW ratios [50,51,52] are linked to insulin resistance, obesity, and metabolic syndrome. Although the adipose mass considerably increases in obesity, adiponectin concentration is strongly reduced in obese patients due to a chronic inflammation of this tissue, mediated by tumor necrosis factor-α (TNF-α), a suppressor of adiponectin expression (Figure 1) [35]. Similarly, in obesity-related disorders, serum adiponectin levels are notably lower in obese subjects, compared to normal-weight subjects. After weight loss, compared to basal levels, adipose tissue functions improve and serum adiponectin levels increase.

During the last decade, serum adiponectin levels have been shown to be modified in patients affected by several diseases; moreover, it is known that adiponectin acts as a tumor suppressor factor and as an inhibitor molecule of the immune system, possesses anti-atherogenic actions, and is implicated in several inflammatory responses [53]. Regarding this function, previous reports describe contrasting results, as adiponectin acts both as a pro- and as an anti-inflammatory molecule [44]. Such a discrepancy is coherent with the evidence that adiponectin exerts different functions depending on many factors, such as targeted tissues/organs and inflammatory state.

## 4. IGF-1 and Adiponectin in Relation to Obesity, Diabetes, and Insulin Resistance

Obesity is a condition characterized by a link between body fatness and risk of different metabolic diseases, including different types of cancers [44]. Recent epidemiological studies provided evidence that insulin, GH/IGF-1, and adiponectin signaling are molecular pathways interconnected with each other and linking obesity to metabolic diseases risk [54]. In a recent cross-sectional study, performed on Chinese nondiabetic obese children and adolescents, low serum IGF-1 levels have been associated with insulin resistance, dyslipidemia, obesity, and the presence of metabolic syndrome [55]. Sirbu et al. found in obese nondiabetic women a significant negative correlation between IGF-1 levels and markers of obesity (BMI and waist circumference) and inflammation (C-reactive protein, CRP), as well as a positive correlation with adiponectin levels [56]. These findings might link liver dysfunction observed in obese patients to their low IGF-1 levels; furthermore, it has been shown that adiponectin is negatively correlated to hepatic insulin resistance and hepatic fat content [57]. Such evidence could explain the direct correlation between the two protein hormones. In the study from Albert et al., it was demonstrated that a low dose of rhGH determines an increase in serum IGF-1 levels and a modest decrease in body fat of obese subjects with functional GHD; on the other hand, the treatment did not produce any change in serum adiponectin, leptin, or CRP, leading the authors to state that the rhGH treatment had no effects on adiposity markers [58]. Differently, Makimura et al. classified their group of obese subjects into normal, GH-sufficient, and GH-deficient via GH stimulation testing, observing in all of them a positive association between peak-stimulated GH and serum adiponectin levels; moreover, the GH-deficient group, compared to the other groups, showed a greater carotid intima-media thickness [59].

Regarding obese patients after weight loss, Pardina et al. reported that the levels of both adiponectin and IGF-1 increased, compared to basal levels, in 34 morbidly obese patients after bypass surgery, even if the two parameters were not correlated [54].

Recently, low levels of adiponectin have been associated with an increased risk of obesity-related cancers and development of more aggressive phenotype, with concomitant alterations in the bioavailability of IGF-1 [60]. In particular, obese women, characterized by hyperactivation of IGFs and dysregulation of adiponectin pathways, seem to be at a higher risk of breast cancer [61].

Different results have been described in type 2 diabetes mellitus (T2DM) populations. Kanazawa et al. reported a significant inverse association between IGF-1 and adiponectin levels in Japanese men affected by T2DM; the correlation was independent of age, duration of diabetes, BMI, and renal function, suggesting that IGF-1 might directly suppress serum adiponectin levels [62]. A clear explanation for the direct correlation between the two hormones is not given, but the authors speculate that causal relationships cannot generally be referred in a cross-sectional study. Further, as most of circulating IGF-1 is bound to specific IGFBPs, able to positively or negatively modulate IGF action, this could be reflected on the regulation of serum adiponectin levels.

The signaling interplay between IGF-1 and adiponectin in relation to insulin resistance is a very complex matter, above all when an abnormal GH secretion is taken into account. As for GHD, in adult patients, under replacement GH therapy, a variety of different conditions have been recorded: some studies show that serum adiponectin levels and insulin resistance are not affected [63,64], others describe unchanged adiponectin values and reduced [65,66] or increased insulin resistance [67], and others report an increase in adiponectin with reduced [68] or unchanged insulin resistance [69]. In GHD children, it has been observed that the lower the IGF-1 bioavailability index obtained from the IGF-1/IGFBP-3 ratio, the higher the adiponectin levels, when compared to GHD children with a high index; moreover, the lower IGF-1 bioavailability index was characterized by lower insulin resistance [70]. As a consequence, the authors found that the better metabolic profile among the two GHD children groups, at a low or high bioavailability index, is essentially related to higher adiponectin secretion [70].

Fukuda et al. analyzed the serum adiponectin levels in adults affected by GHD or active acromegaly in comparison with healthy age-matched subjects (control group) [71]. They observed that adiponectin concentration in the control group did not differ from GHD or acromegalic patients’ values; however, the authors found, in GHD, adiponectin levels statistically lower than in acromegalic patients matched for BMI [71]. Moreover, adiponectin levels were inversely related to serum IGF-1 in acromegaly, suggesting that both IGF-1 and BMI are determinant factors affecting circulating adiponectin levels in patients with active disease. On the contrary, in GHD, serum adiponectin levels were inversely related to BMI but positively to insulin sensitivity; such a finding led the authors to speculate that in GHD the increased insulin resistance could be due to a high fat mass and not to the hormone deficiency, whereas, in acromegaly, it could be a direct consequence of GH excess [71]. Additionally, Lam et al. suggested that hyperinsulinemia is the link between GH excess and hypoadiponectinemia in patients with active acromegaly: in particular, insulin resistance may be a consequence of GH/IGF-1 overproduction and a cause of reduced adiponectin expression [72]. Conversely, Silha et al. observed a significant increase in serum adiponectin levels and a modest increase in insulin resistance within a small group of obese patients with active acromegaly [73]. The authors ascribed the acromegaly-dependent insulin resistance to two counteracting factors: the GH-mediated lipolysis, determining an increase in free fatty acids, and a simultaneous compensatory mechanism performed by adiponectin, enhancing insulin sensitivity. An increase in serum adiponectin levels, together with an improved insulin sensitivity, was reported by Wiesli et al., who analyzed the effect of pituitary surgery in acromegalic patients [74]; such findings led to the hypothesis that factors able to interfere with adiponectin expression counteract each other when GH is overexpressed. Further, Ronchi et al. reported unchanged serum adiponectin levels between patients with active acromegaly and controls [75]; moreover, they found no correlation between adiponectin expression and several metabolic (BMI, waist circumference, and insulin resistance) and/or cardiovascular factors (lipid profile and blood hypertension) in acromegalic patients [75]. According to the authors, the missed reduction in adiponectin expression may be an indirect hint of a possible involvement of the GH/IGF-1 axis on the adipose tissue [75].

A hallmark of active acromegaly is an alteration of the distribution of the adipose tissue, mainly characterized by a decrease in fat mass, an increase in lean mass, and an ectopic extra-adipose tissue deposition [76,77,78]; such a condition determines an impaired insulin action in both hepatic and extra-hepatic tissues [79]. In this regard, Olarescu et al. reported an elevated metabolic activity for adipose tissue in acromegaly, able to affect the production and the secretion of adipokines [76,77,78]. As for adiponectin, data are controversial in active acromegaly, as quoted above, but recently White et al. proved the negative regulator effect of STAT5A transcription factor on the adiponectin expression in murine 3T3-L1 preadipocytes after GH treatment [80].

The adipose tissue function and distribution affect not only insulin resistance but also cardiovascular (CV) risk. In this regard, a visceral adiposity index (VAI) has recently been proposed as a surrogate marker of adipose tissue dysfunction associated with both metabolic syndrome and CV risk [81]. Ciresi et al. evaluated such an index in active acromegaly in relation to serum adiponectin levels by dividing the patients into normal and high VAI groups [82]. VAI strongly associated with patient age and GH/IGF-1 levels indicating that older patients having higher hormonal levels show a more severe visceral adipose dysfunction. Moreover, compared to patients with a normal VAI, high VAI patients had higher GH and IGF-1 values, and a lower insulin sensitivity and adiponectin concentration, suggesting proneness to metabolic syndrome and CV risk [82].

## 5. IGF-1 and Adiponectin in Relation to Cardiovascular Diseases

There is considerable evidence that the modulation of GH/IGF-1 axis has implications on the CV system [31]. In vitro and in vivo studies demonstrated the hypertrophic effect of IGF-1; moreover, IGF-1 prevents myocardial apoptosis and increases cardiac contractility by inducing an intracellular calcium influx upon cardiac IGF-1R activation [83,84]. The alteration of GH/IGF-1 signaling, caused by an excessive or a defective GH secretion, is associated with CV impairment both in GHD and in acromegaly [31,85]. There are also data linking low serum IGF-1 levels to an increased risk of CV diseases, including coronary artery and ischemic heart diseases, myocardial infarction, heart failure (HF), and stroke [84]. These associations, however, revealed a very complex interplay between IGF-1 and CV systems, and conflicting results have been reported in clinical studies [86,87,88]. The reasons behind such controversial data could be partly explained by the extreme variability of the proposed study design, such as patient recruitment differing in age and sex and eventually in co-morbidities, or the estimation of IGF-1 bioavailability by applying different in vitro biochemical assays [89]. Certainly, all the evidence, even if controversial, supports that the CV system is a target of the IGF-1 action. In this context, taking into account the role of adiponectin, a correlation between its high serum levels and its protective effect against CV disease has been reported [89], acting as an anti-inflammatory and anti-atherosclerosis protein hormone in a dose-dependent manner [90]. Contrary to IGF-1, in vitro and in vivo studies reported an anti-hypertrophic myocardial effect of adiponectin that may improve cardiac remodeling in pathological conditions [91,92].

As for the IGF-1/adiponectin relationship in CV, Watanabe et al. analyzed the association between serum IGF-1/IGFBP-3 ratio and adiponectin levels in more than 100 HF patients with left ventricular systolic dysfunction compared to control subjects [93]. The authors found a significant inverse correlation of IGF-1 axis with adiponectin or the cardiac biomarker B-type natriuretic peptide (BNP). In fact, the IGF-1/IGFBP-3 ratio is reduced in patients, especially in conditions of severe HF [93]. One might suggest that low IGF-1 in HF patients with poor prognosis may influence the high secretion of adiponectin. However, in this speculation, it is important to take into account the IGF-1 bioavailability index, which has a significant impact on metabolic profile [70,94]. When the relationship of cardiac parameters with IGF-1 or adiponectin was explored in patients with severe obesity, Sirbu et al. reported the negative association of adiponectin and the positive association of IGF-1 with the left ventricular mass (LVM) [95]. On the other hand, in obese adolescents, it has been recently reported that IGF-1 and IGFBP-1 are negatively associated with traditional CV disease biomarkers and IGFBP-1 is positively associated with adiponectin [96].

Considering CV risk in acromegaly, Verhelst et al. analyzed two specific markers of CV risk among acromegalic patients, subdivided into a controlled disease group (−2 < IGF-1 Z-score < +2) and an active disease one (IGF-1 Z-score > +2) [97]. They took into account highly sensitive CRP (hs-CRP), related to CVD and the *N*-terminal pro-BNP (NT-proBNP), diagnostic for congestive heart failure [97]. The authors found low levels of hs-CRP in active acromegalic patients compared to control subjects, indicating a lower CV risk in active disease, whereas high NT-proBNP values were reported in controlled patients compared to healthy subjects, suggesting a controversial role for such a marker. Serum adiponectin levels did not change between the two patient groups [97].

Additionally, Gurbulak et al. explored serum adiponectin levels in relation to cardiac parameters in acromegalic patients [98]. Serum adiponectin levels were higher in acromegalic patients compared to healthy controls, whereas they did not change between VAI groups; moreover, adiponectin showed a positive correlation with the LMV index, while VAI was positively associated with LVM. According to the authors, adiponectin values are more suitable to evaluate active vs. inactive disease; conversely, VAI could be useful in assessing cardiac risk in acromegaly [98].

Despite this evidence, further studies are required to elucidate the molecular mechanisms underlying the IGF-1/adiponectin interplay in pathological conditions leading to CV diseases.

## 6. IGF-1 and Adiponectin in Relation to Cancer

As previously stated, both IGF-1 and adiponectin affect glucose and fat metabolism; hence, the signaling of two hormones could cross and interfere with the energy metabolism of a specific tissue/organ, healthy or transformed one. Such energy-related mechanisms appear more active in pre-neoplastic or neoplastic cells, supporting the high-energy requirement due to increased proliferation [99].

It is well known that plasma IGF-1 and IGF-1R concentrations regulate the growth and survival of neoplastic cells by activating PI-3K, Akt, the mTOR complex, and the MAPK pathways (Figure 1) [100]. These complex signal transduction pathways, in fact, are commonly activated in epithelial cancers, together with kidney and breast cancer [101,102]. This cascade of intracellular signals overlaps the insulin pathways; interestingly, high serum insulin levels increase IGF-1 synthesis in the liver with a simultaneous reduction of the production of IGFBPs, especially IGFBP-1 and 2. Such an effect promotes IGF-1 bioactivity that, in turn, triggers cell growth signaling through IGF-1R increased expression [103,104]. Accordingly, serum IGF-1 levels have been linked to a higher risk of certain types of cancer, including breast, colorectal, and prostate cancer [105].

To date, the anti-proliferative and tumor suppressor role of adiponectin remains elusive. Serum adiponectin levels are inversely associated with tumor cell growth [106]. Mauro et al. reported that in breast cancer such low levels promote cell growth by adiponectin/AdipoR1 and IGF-1/IGF-1R activation pathways [60]. Other recent findings suggest that the anti-tumorigenic effect of adiponectin becomes evident only in association with other GFs such as insulin and IGF-1, leptin and inflammatory cytokines as IL-6 and TNF-α (Figure 1) [106,107,108]. In general, low adiponectin plasma levels, present in obese subjects, have been associated with an increased risk of different types of cancer such as endometrial, breast, and colorectal cancer [109,110,111,112,113]. Molecular mechanisms underlying these effects have been previously described in the granulocyte cells, where adiponectin serum concentration influences tyrosine phosphorylation of the IGF-1R-β subunit and activates MAPKs [114]. A similar mechanism was also proposed in breast cancer tumorigenesis, where the role of adipokines seems to be dependent on tumor phenotype [115]. In fact, adiponectin inhibits cell proliferation in human estrogen receptor α negative (ERα^−^) breast cancer cells [116,117,118], whereas data for ERα^+^ cells are confounding ([60] and references therein). The authors demonstrated that adiponectin is able to transactivate ERα in a ligand-independent manner, and that this event is a prerequisite for adiponectin-induced MAPK activation, which mediates proliferation and inhibition of apoptosis. Furthermore, it has been shown that low concentrations of adiponectin rapidly increase IGF-1R phosphorylation in ERα^+^ breast cancer cells, and that the use of ERα siRNA prevents this effect. In turn, a specific siRNA for IGF-1R prevents adiponectin-induced ERα transactivation, thus proving the existence of a functional interplay among adiponectin/AdipoR1, ERα, and IGF-1R, promoting ERα^+^ breast cancer cell growth [60,115].

Increased insulin and IGF-1 levels, together with decreased adiponectin levels, are also involved in the development of colon cancer.

While it has been demonstrated that increased IGF-1 serum levels contribute to tumor development and progression in colon cancer cells [119,120], the association between serum adiponectin concentration and the increased risk of colon cancer, carcinogenesis, or cancer progression has not been completely elucidated until now [121]. Controversial results have been reported so far on the association between serum adiponectin and increased risk of colorectal adenoma. Some cross-sectional as well as meta-analysis studies suggest that low serum adiponectin levels are related to an increased risk of colorectal adenoma [122,123,124], whereas Ochs-Balcom et al. reported no correlation between serum adiponectin levels and an increased risk of colorectal and adenoma in Caucasian and African-American male subjects [125]. In general, serum levels of markers associated with obesity, such as insulin, IGF-1, IL-6, TNF-α, leptin, and adiponectin, may trigger the PI-3K/Akt signaling pathway to promote cell growth and colon cancer carcinogenesis; conversely, the adiponectin-mediated inhibition of PI-3K/Akt signaling pathway, through the suppression of TNF-α and IL-6, is able to regulate cell growth inhibition (Figure 1) [126].

Circulating IGF-1, adiponectin, and others (IGFBP-3, HMW adiponectin) were also investigated in relation to renal cell carcinoma (RCC) risk, in a prospective study within the Prostate, Lung, Colorectal and Ovarian (PLCO) Cancer Screening Trials (a population-based multi-center randomized screening trials in the US) [127]. Data, however, did not provide definite evidence of the starting hypothesis, although the authors observed a weak association between adiponectin and IGFBP-3. In particular, high adiponectin and HMW adiponectin levels were related to a reduced RCC risk; on the contrary, high IGF-1 and IGFBP-3 levels suggestively increased RCC risk [127].

In general, more studies are hence needed to confirm the putative relationship between the hormones’ profile patterns and cancer risk.

## 7. Conclusions

Despite the growing experimental evidence, here reported, supporting the functional interplay between IGF-1 and adiponectin, furthering the knowledge of such a relationship is a mandatory perspective for future studies. Indeed, more insights are needed to clarify their concerted role in obesity, diabetes, insulin resistance, HF, and cancer. In fact, the two hormones have been reported as both inversely and directly correlated within the same pathological context. The reasons behind these conflicting results are at present not fully elucidated because several gaps leave open questions at both the molecular and physiological levels. Among them, for example, the focus could be directed toward the complexity of the GH/IGF-1 system, consisting of GH, IGF-1, different binding proteins, and co-regulatory proteins; in such a system, several assays for the measurement of the unbound active fraction of IGF-1 have been proposed and applied, complicating the jigsaw [89]. Another missing piece of information is related to the role played by the IGFBPs with respect to the adipose tissue, through their IGF-dependent and/or IGF-independent actions ([128] and references therein). As for adiponectin, it is not known if and how the several circulating adiponectin oligomeric isoforms, from low to very high MW, could contribute differently to the modulation of the GH/IGF-1 system.

In conclusion, the current knowledge strongly encourages further research into the common, as well as novel, mechanisms through which IGF-1 and adiponectin exert their concerted action.
